# Virgin queen attraction toward males in honey bees

**DOI:** 10.1038/s41598-017-06241-9

**Published:** 2017-07-24

**Authors:** Florian Bastin, Hanna Cholé, Grégory Lafon, Jean-Christophe Sandoz

**Affiliations:** Evolution, Genomes, Behaviour and Ecology, CNRS (UMR 9191), Univ Paris-Sud, IRD, Université Paris-Saclay, 1 avenue de la Terrasse, 91198 Gif-sur-Yvette, France

## Abstract

Although the honeybee is a crucial agricultural agent and a prominent scientific model organism, crucial aspects of its reproductive behaviour are still unknown. During the mating season, honeybee males, the drones, gather in congregations 10–40 m above ground. Converging evidence suggests that drones emit a pheromone that can attract other drones, thereby increasing the size of the congregation. Virgin queens join the vicinity of the congregation after it has formed, and mate with as many as 20 males in mid-air. It is still unclear which sensory cues help virgin queens find drone congregations in the first place. Beside visual cues for long-range orientation, queens may use olfactory cues. We thus tested virgin queens’ olfactory orientation on a walking simulator in which they have full control over odour stimulation. We show that sexually-mature virgin queens are attracted to the odour bouquet from a group of living drones. They are not attracted to the bouquet from a group of workers. In addition, non-sexually receptive females (workers) of the same age are not attracted to the drone odour bouquet. Interpreted in the context of mating, these results may suggest that virgin queens use volatile olfactory cues from the drones to find the congregations.

## Introduction

Bees (Hymenoptera: Apoidea: Anthophila) represent more than 20,000 species, from solitary to eusocial, which display a wide spectrum of mating behaviours^1–3^. Some species mate at the female emergence site (e.g. *Colletes cunicularius* ^4^ and *Centris pallida* ^5^), others at resource-based sites (*Anthidium manicatum* ^6^), while still others use nuptial flyways (*Apis* species^7^). Despite this diversity of mating strategies, many bee species are known to use sex pheromones for reproduction^2^. Sex pheromones are defined as odours, produced by either males or females, which stimulate behavioural reactions and/or induce physiological effects in the opposite sex, bringing the sexes together for the purpose of mating^2^. Bees have large glandular systems allowing both females and males to produce sex pheromones which attract conspecifics. Female exocrine secretions from the mandibular glands are the most common bee sex pheromones. They induce male attraction in some Colletidae^4^, Andrenidae^8^ and Apidae like carpenter bees^9^, stingless bees^10^, bumble bees^11^ and honey bees^12^. In some cases, the male secretions produced by the mandibular glands or the labial glands are also used to mark spots along male flight paths. These spots are attractive to females but also to conspecific males in some Andrenidae^13^, and Apidae, like carpenter bees^9^, bumble bees^14^ and orchid bees^15^.

The importance of male-produced pheromones for bee reproduction has long been underestimated, probably because they were less intensively studied. Even in some well-studied species their existence and role are still unclear. A good example is the honeybee *Apis mellifera*, a worldwide economically valuable pollinator and a main-stream scientific model in diverse fields such as genetics, physiology, ethology, neurobiology and animal cognition^16–23^. Honeybees are eusocial insects, characterized by a reproductive division of labor between one fertile female, the queen and thousands of facultatively sterile females, the workers^17^. Honeybees display a particularly striking mating behaviour^24–30^. During the mating season, and on favourable weather conditions, honeybee males, the drones, fly out and gather high in the air at discrete congregation areas located usually 10–40 m above ground, with a diameter of 30–200 m^7, 31–33^. These drone congregations can contain as many as 11,000 drones from up to 240 different colonies^33–36^. Then, about one hour after the peak of drones’ departure, virgin queens leave the hive and fly to the vicinity of the drone congregation^7, 25, 37^. As soon as a virgin queen is present, many drones are attracted to her, both by olfactory signals (the mandibular gland sex pheromone, 9-oxo-2-decenoic acid, 9-ODA) and by visual cues at shorter range^12, 38^. Drones follow the virgin queen in a comet-like swarm and engage in a scramble competition, each individual struggling for the most promising position to approach and mate with the queen^38^. Within 15–30 min, the queen mates with 10–20 drones, which die directly after copulation^35, 39, 40^. After one or two nuptial flights, the queen returns to the colony and after a delay of a few days, starts laying eggs^30^.

Because of obvious limitations related to the low accessibility of drone congregations located high in the air, the exact cues used by drones and virgin queens to find them are still unclear. Even though the life span of a drone is limited to a few weeks^41^, drone congregation areas are surprisingly constant in location from year to year, and some congregations have been reported to form consistently at the same place over decades^7, 42–44^. Although small “artificial” congregation areas can be elicited with large amounts of queen pheromone^45^, it is clear now that the presence of a queen is not necessary for forming a drone congregation and, as indicated above, queens usually arrive after the drones^7, 25, 37^. Visual cues on the horizon, such as mountains, valleys and tree tops in less mountainous regions appear to be used for long-range orientation^27, 31, 44, 46^. In addition to visual cues, anomalies in terrestrial magnetic field have been proposed to play a role in drone congregations^47^. However, horizon cues or magnetic field cannot explain short-range orientation at the area itself and the clear-cut dimensions of a drone congregation: when a virgin queen leaves the congregation area, drones rapidly stop their pursuit and return to their consexuals in the congregation^33, 37, 44^. To explain these observations, a drone-produced pheromone that would attract other drones has been proposed several decades ago^34, 48, 49^. However, due to the difficulties of testing these ideas in natural mating conditions, research in this direction has little progressed since then. Recently, we tested this hypothesis in the laboratory by using tethered drones freely-walking on an air-suspended trackball^50^. Our study demonstrated that honeybee drones are indeed attracted to the odour bouquet from a group of drones^50^. These data clearly suggest that a drone-produced attractive odour substance may be involved in the formation of drone congregations. If this is the case, an especially interesting possibility is that virgin queens may also use such a cue when orienting toward drone congregations. In the present study, we thus explored the olfactory preferences of virgin queens under controlled experimental conditions using our walking simulator^50^. As a preliminary step, we demonstrated with an olfactory information transfer experiment on workers that the attractiveness of an odorant is translated in the walking simulator by an increase in the time spent and in the distance travelled by the animal in the odour (Supplementary Experiment 1). Then, we show that honeybee virgin queens are indeed attracted to the odour bouquet from a group of drones. Our data demonstrate that this attraction is both emitter- and receiver-specific: it is only displayed toward drone odours, not toward worker odours. In addition, this behaviour is elicited in sexually-receptive females (virgin queens) but not in sterile females (workers). Even if these data need to be confirmed in a natural mating context, they could suggest that drone-produced odours may constitute useful cues for virgin queens to find drone congregations.

## Results

**Figure 1:**
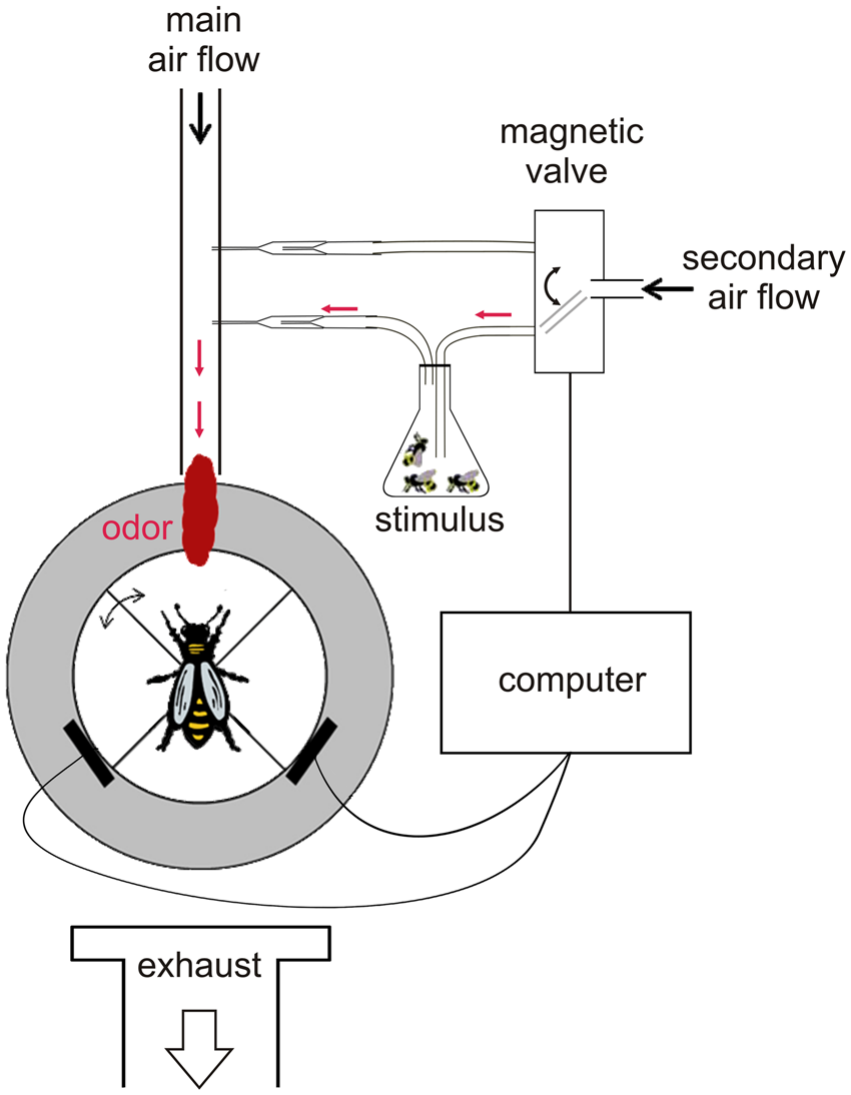
Walking simulator setup. A tethered honeybee queen or worker is allowed to freely walk on an air-supported ball (in white). The bee can easily turn the ball under herself. Ball displacement is recorded via two computer-mouse sensors (black bars close to the ball), which allow reconstructing the bee’s walking path. Odour stimulation is provided via a main, constant, air-stream directed to the bee. Odours are quickly removed from the setup by an exhaust behind the honeybee. All experiments were conducted in complete darkness. The ball is virtually divided into 4 quadrants, one of which is designated as the odour quadrant. After an accommodation phase of 5 min (‘before’ phase), stimulus control is granted to the bee for 5 min (‘during’ phase): whenever the bee’s virtual heading is in the odour quadrant (as shown on the figure), odour stimulation is activated using the computer-controlled magnetic valves. This allows quantifying, whether the animal preferred receiving odour stimulation or not. Groups of 10 living drones or workers were used as stimuli.

The olfactory preference of honey bees was tested on a walking simulator in the dark. Bees were allowed to freely walk on a track ball, which they could easily turn under them (Fig. 1). The ball was divided in four virtual quadrants, one of which was pseudorandomly designated as the odour quadrant. After a stimulation-free accommodation phase of 5 min (henceforth termed ‘before’ phase), stimulus control was granted to the bee for 5 min (henceforth termed ‘during’ phase). Whenever the bee was heading toward the odour quadrant, odour stimulation was activated and delivered directly in front of the bee to its antennae. In a preliminary experiments, we first showed that after an appetitive conditioning experience (conditioning of the proboscis extension response, PER^51–53^), workers spent more time and travelled a longer distance heading toward the quadrant dispensing the learned odour (see Supplementary Experiment 1). This result reproduces the clear attraction of conditioned honey bee workers toward a learned odour, which was already observed in a variety of experimental situations: walking in a four-armed olfactometer^54^, walking in a Y-maze^55^ or flying in a wind tunnel^56^. Thus, the walking simulator allows measuring olfactory attraction in honey bees.

### Experiment 1: Virgin queen attraction toward drones’ odour bouquet

**Figure 2:**
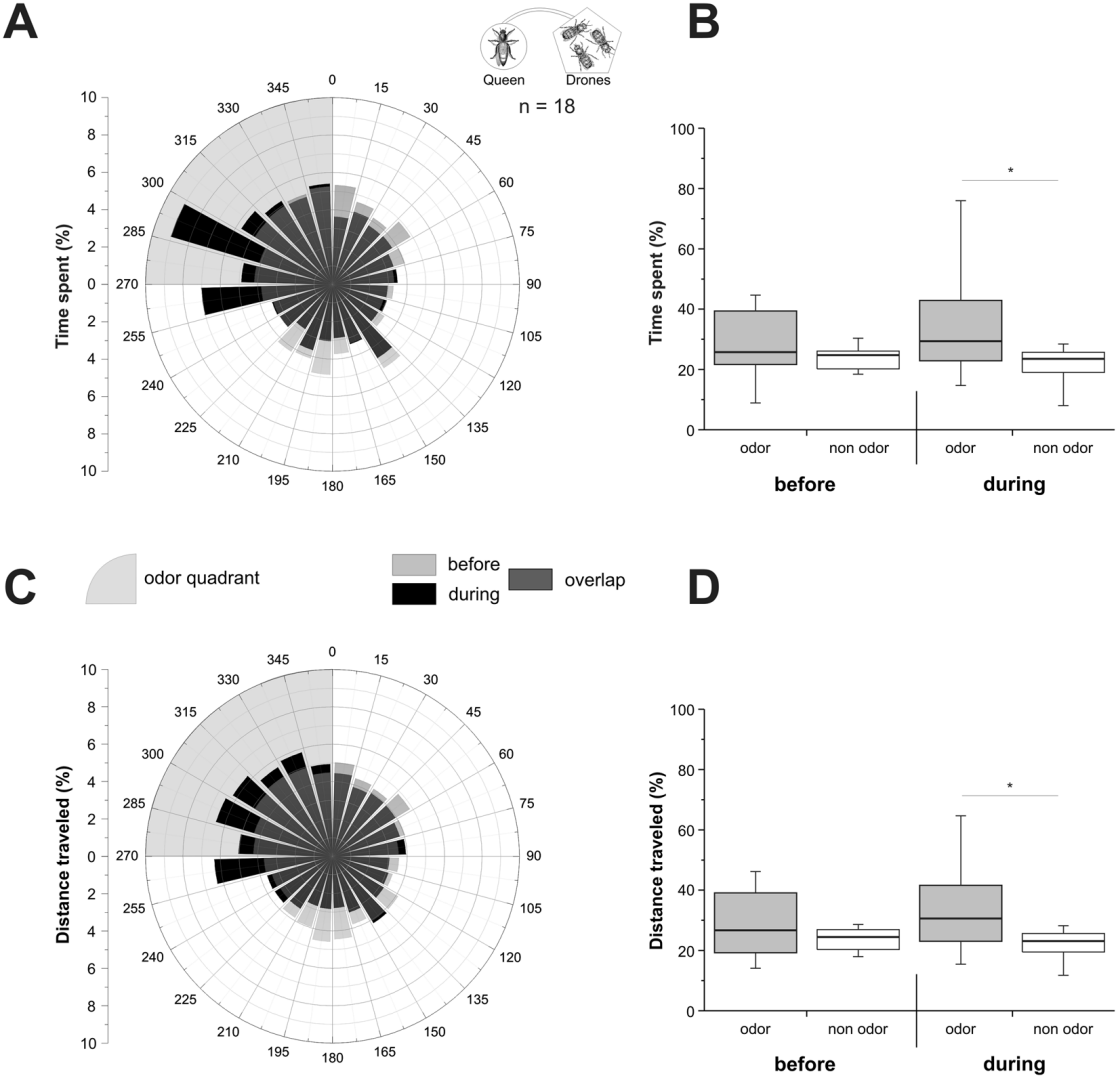
Experiment 1: virgin queens’ response to drone odour bouquet. Virgin queen’s behaviour on the walking simulator, when stimulated with the odour bouquet of 10 drones. (**A**,**C**) Circular histograms showing the percentage of time spent (**A**) or of distance travelled (**C**) by virgin queens according to 15° sectors, with the odour quadrant being represented on the upper left (grey area). Light grey bars represent the 5 min before odour stimulation (‘before’), black bars represent the 5 min during stimulation (‘during’), and hence, dark grey bars show the overlap of the two phases. (**B**,**D)** Histograms of the percentage of time spent (**B**), or of distance travelled (**D**) by virgin queens in the odour quadrant (gray box) and on average in the three odourless quadrants (white box) before and during odour stimulation. *p < 0.05, Wilcoxon matched pairs tests.

The olfactory preference of sexually-mature honey bee virgin queens toward the odour bouquet from a group of 10 living drones was tested in the walking simulator. In the accommodation phase (‘before’), the time spent (Fig. 2A,B) as well as the distance travelled (Fig. 2C,D) by the virgin queens were homogenous between the odour quadrant and the non-odour quadrants (Wilcoxon test, Z_time_ = 1.07, p_time_ = 0.29; Z_distance_ = 1.11, p_distance_ = 0.27). However, in the odour stimulation phase (‘during’), virgin queens’ behaviour changed, and they oriented toward the odour quadrant, receiving the effluent bouquet from a group of 10 drones. Thus, virgin queens spent significantly more time (Fig. 2B), and travelled longer distances (Fig. 2D) in the odour quadrant compared to the non-odour quadrants (Z_time_ = 2.05, p_time_ = 0.041; Z_distance_ = 2.11, p_distance_ = 0.035). This experiment shows that virgin queen bees are attracted to volatile molecules produced by drones.

### Experiment 2: Is virgin queen olfactory attraction expressed toward all conspecifics?

**Figure 3:**
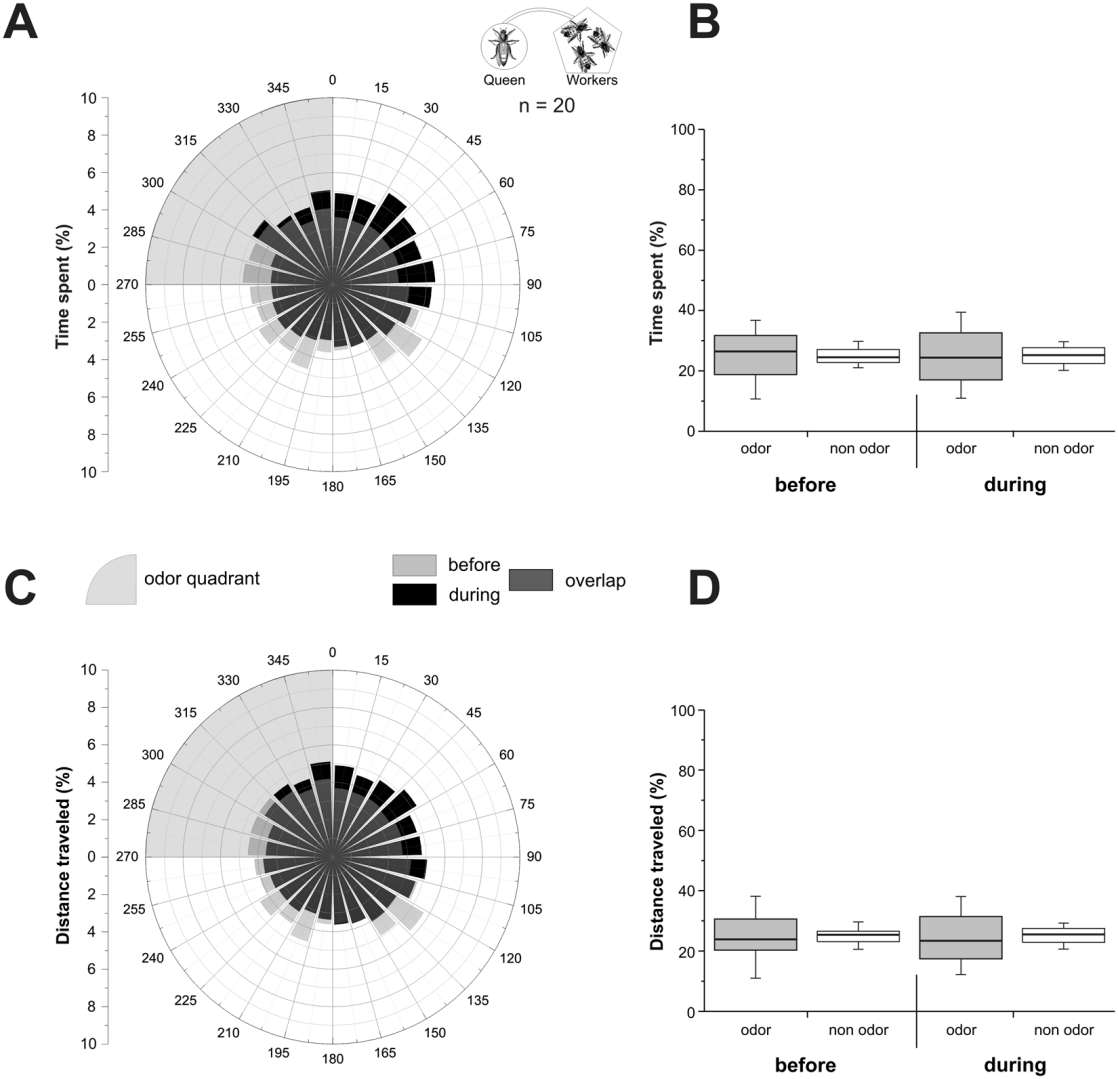
Experiment 2: virgin queens’ response to worker odour bouquet. Virgin queens’ behaviour on the walking simulator, when stimulated with the odour bouquet of 10 workers. (**A**,**C**) Circular histograms showing the percentage of time spent (**A**) or of distance travelled (**C**) by virgin queens according to 15° sectors, with the odour quadrant being represented on the upper left (grey area). Light grey bars represent the 5 min before odour stimulation (‘before’), black bars represent the 5 min during stimulation (‘during’), and hence, dark grey bars show the overlap of the two phases. (**B**,**D**) Histograms of the percentage of time spent (**B**), or of distance travelled (**D**) by virgin queens in the odour quadrant (gray box) and on average in the three odourless quadrants (white box) before and during odour stimulation.

Next, we tested the specificity of virgin queens’ olfactory attraction toward drones. We asked whether a similar attraction would be expressed toward the odour bouquet from any other conspecifics. A new set of virgin queen bees was thus tested with the odour bouquet from a group of 10 workers. Circular repartition of the time spent (Fig. 3A) and the distance travelled (Fig. 3C) by the virgin queens was quite homogenous before but also during the odour stimulation phase. Accordingly, the time spent (Fig. 3B), and the distance travelled (Fig. 3D) by virgin queens was not significantly different between odour and non-odour quadrants, before stimulation (Z_time_ = 0.41, p_time_ = 0.68; Z_distance_ = 0.26, p_distance_ = 0.79) and during stimulation (Z_time_ = 0.04, p_time_ = 0.97; Z_distance_ = 0.26, p_distance_ = 0.79). The odour bouquet from workers was therefore not attractive to virgin queens. This suggests that virgin queens’ olfactory attraction is specific to drones’ odour bouquet.

### Experiment 3: Is virgin queens’ olfactory attraction specific to their sexual motivation?

**Figure 4:**
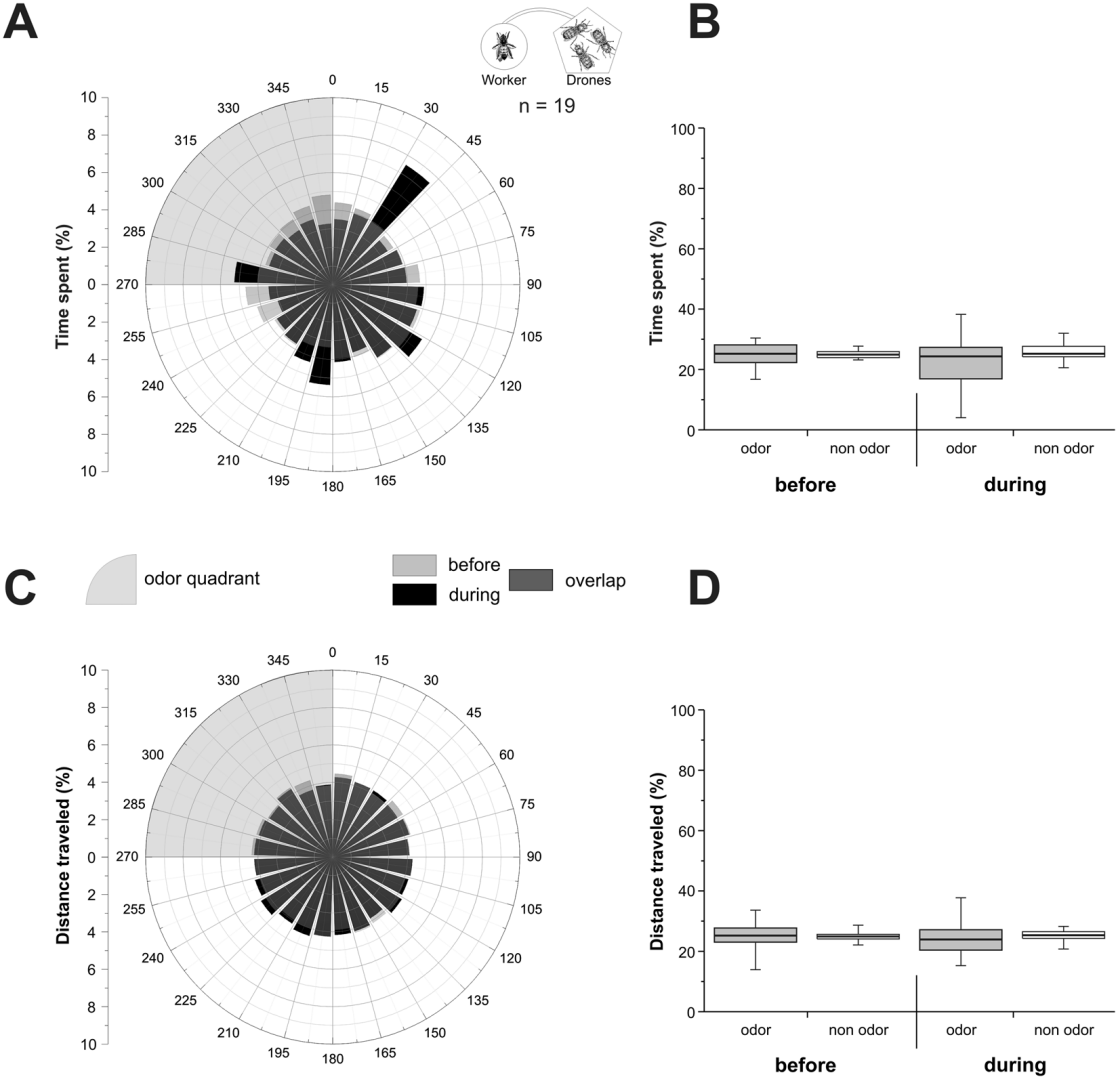
Experiment 3: workers’ response to drone odour bouquet. Workers’ behaviour on the walking simulator, when stimulated with the odour bouquet of 10 drones. (**A**,**C**) Circular histograms showing the percentage of time spent (**A**) or of distance travelled (**C**) by workers according to 15° sectors, with the odour quadrant being represented on the upper left (grey area). Light grey bars represent the 5 min before odour stimulation (‘before’), black bars represent the 5 min during stimulation (‘during’), and hence, dark grey bars show the overlap of the two phases. (**B**,**D**) Histograms of the percentage of time spent (**B**), or of distance travelled (**D**) by workers in the odour quadrant (gray box) and on average in the three odourless quadrants (white box) before and during odour stimulation.

We then determined whether the observed attraction of virgin queens toward drone volatiles is related to their being a reproductive and sexually-motivated female, or if all female honey bees behave similarly. We consequently tested the behaviour of worker bees toward the odour bouquet from a group of 10 living drones. These workers had the same age as the virgin queens tested in the previous experiments. Circular repartition of the time spent (Fig. 4A) and the distance travelled (Fig. 4C) by workers was homogenous both before and during the odour stimulation phase. The time spent (Fig. 4B), and distance travelled (Fig. 4D) by workers was not significantly different between stimulated and non-stimulated quadrants both before stimulation (Z_time_ = 0.24, p_time_ = 0.81; Z_distance_ = 0.08, p_distance_ = 0.94) and during stimulation (Z_time_ = 0.93, p_time_ = 0.35; Z_distance_ = 0.28, p_distance_ = 0.78). The bouquet from living drones did not induce any change in workers’ behaviour. We conclude that attraction to drone volatiles is specifically expressed by sexually-receptive females.

### Castes difference in travelled distance

On average, there was a significant heterogeneity among the distances travelled in 10 min by the bees in the three experiments (Kruskal-Wallis test, H = 6.48, p = 0.039; Supplementary Fig. 4). The distance travelled by virgin queens was not different when queens were tested with 10 drones or 10 workers, (first and second experiment respectively, Dunn’s multiple comparisons, q = 0.45, p = 1.00). However, workers tested with the drone odour bouquet (third experiment) travelled a significantly longer distance (8809 ± 1978 mm) than queens tested with workers (second experiment, q = 2.41, p = 0.047), but not significantly longer than queens when drone odour was available (first experiment, q = 1.91, p = 0.17). Odour stimulation did not change the distance travelled by queens when tested with drone odour (first experiment, Wilcoxon matched pairs test, Z = 1.76, p = 0.08) or by workers when tested with living drones (third experiment, Z = 1.45, p = 0.15). Queens however walked significantly less in the period during which the odour from living workers was available than before (second experiment, Z = 3.43, p = 0.0006).

## Discussion

In this study, we determined the odour preferences of honeybee virgin queens under controlled experimental conditions, using the odour bouquet from groups of living conspecifics as stimulation. In our walking simulator, virgin queens were attracted to volatiles emitted by a group of living drones, but did not react to a group of workers. We then demonstrated that attraction toward drone volatiles is specifically expressed by sexually-receptive females, since same-age workers were not attracted to the drone odour bouquet. This is the first evidence in honey bees that drones produce an odour cue that is attractive to sexually-mature virgin queen bees.

In Experiment 1, virgin queens spent significantly more time and travelled a longer distance in the drone odour quadrant when stimulus control was granted to them (i.e. ‘during’ phase). Our system was designed to provide clear criteria for measuring whether or not a bee is attracted to an odour and to allow maximal control over experimental conditions^50^. Indeed in the preliminary experiment, appetitively conditioned bees clearly chose to remain in the odour quadrant and walked longer distances in this quadrant compared to control bees (Supplementary Experiment 1). This behaviour is typical of the olfactory attraction shown by bees toward an odorant with a positive hedonic value^54–56^. Because the bee has full control over odour delivery during the stimulus control phase, it can choose to remain or not in the odour quadrant. Leaving the odour quadrant only means for the animal turning the ball by at most 90°, which is an easy task. Thus, we interpret the time spent in the odour quadrant, as well as the distance travelled in this quadrant, when they are above chance, as indications of a genuine olfactory attraction.

Because of its location in the laboratory, our experimental procedure cannot provide the exact context in which young virgin queens usually depart for their mating flights. We attempted to control experimental variables to approach such a nuptial context. Virgin queens were tested only during the afternoons, on days when drones naturally exited the hives, with mature flying drones, and the queens’ age was controlled (7–15 days) to ensure that they were sexually mature. However, the insects were walking, not flying, and since we wanted to test olfactory attraction specifically, the experiments were performed in the dark. Therefore, we could not provide the bees with the level of multimodal sensory information they receive in a natural mating context. This being said, these controlled experiments may still reveal an attraction of queens toward drones, which could also be manifested during nuptial flights. We will provide interpretations along this line below.

In Experiment 2, we showed that virgin queens are not attracted to the odour bouquet from a group of living workers, confirming that the queen attraction observed in Experiment 1 depends on the sex of the emitter. While interactions between queens and drones are rare within the hive^17^, interactions between queens and workers are numerous. These interactions are however almost exclusively induced by workers^57, 58^. For instance, the queen mandibular pheromone elicits the retinue behaviour, in which workers are attracted to the pheromone, lick, antennate and feed the queen^59, 60^. Concerning virgin queens, they are actively pursued and harassed by workers in the period after swarming, when queen fights take place, eventually inducing surviving queens to leave for mating flights^30, 61, 62^. To conclude, the lack of attraction of our virgin queens to workers could thus have been expected. We observed however that queens reduced their walking speed during the phase when worker odour was presented. While there was a general tendency in all groups for a slight reduction of walking speed in the course of the experiment, the decrease was stronger and significant in this case. One explanation could be that worker odour conveyed a within-hive context to the queen, a situation in which movement speed is reduced compared to outside the hive. Even though our experiments were performed in the dark, drone odour would not provide such an in-colony context. Even during the mating season, a honeybee colony contains many more workers than males (about 50000 workers vs at most 2000 drones^30^). The colony odour is thus more closely related to that of workers than to that of drones.

In Experiment 3, we found no attraction of workers to the odour bouquet from a group of drones. This confirms that not any 7–15 day-old female bee is attracted to drone odour, but rather sexually-motivated females. In nature, interactions between workers and drones are sparse and take place only inside the hive. Workers mostly feed drones by trophallaxis when they are young (until 6–7 days^63^), but also chase them away in case of scarce resources or before winter^64, 65^. Our experiments took place during the reproduction season, and given that the drones used as stimulation are mature and in a similar high satiety state as workers, and as worker’s objective is not reproduction, it seems logical that no attraction toward the drones was expressed by workers.

If our findings apply in a natural mating context, one may propose the following tentative scenario. Drone congregation areas are constant in location from year to year^7, 42–44^. As a first step, drones would follow definite flight lanes toward depressions on the horizon, between prominent landmarks such as mountains or high tree tops^27, 31, 37, 44, 46^. Drone congregations are known to form preferably at intersections and branching points of these flyways^33^. One possibility is that a reduced flight speed when drones reorient at these intersections would slightly increase their numbers locally, and at this moment, olfactory sensory information may take over. Since drones are attracted to the odour bouquet of other drones^48–50^, they would accumulate at the intersections and branching points. Their odour bouquet would build up and by a snowballing effect, more and more drones may be attracted to this location, resulting over time in the formation of a drone congregation in this area. It has been shown that a minimum number of drones is needed to stabilize a congregation (more than 1000)^30^. Queens arrive in the vicinity of drone congregations ~1 h after the drones^7, 25, 37^, i.e. when drone congregations are already well-defined, and possibly with strong drone-produced odours. Although virgin queens can in principle follow the same visual cues as the drones when they start their mating flights, the drone-odour attraction we demonstrated could allow queens to locate congregations more quickly. One should contemplate that it is crucial for virgin queens to achieve optimal flight durations to avoid increased predation and weather risks^66–68^. Indeed a considerable number of queens do not return to the colony from their nuptial flights indicating a significant mating risk^18^. Queen’s use of drone-produced volatile emissions could in principle be selective because it would increase their probability of quickly finding a congregation and being successfully mated. It should be noted that while queen attraction was moderate (although significant) in our experiments, they received the effluent from only 10 drones. In nature, drones congregation may contain as many as 11,000 drones^34–36^, which could greatly enhance the strength of the attraction and the distance from which attraction starts.

While the attractive nature of drone volatile emissions is established on drones^48–50^ and now on virgin queens, the production site of the attractive odour is still unknown. In numerous genera of ants and bees, the males’ mandibular glands have been suggested as the source of sex attractants, although in most cases the active components have not yet been conclusively identified^2^. In honeybees, Gerig showed that drones’ crushed heads, proposed to a congregation, were attractive for other drones^48^. Later, Lensky *et al*. proposed drones’ mandibular glands as a possible source for the putative drone-produced attractive signal because they observed drone attraction in a congregation to an extract of 50 drone mandibular glands^49^. A major role of the drones’ mandibular glands is debated though, because they are greatly reduced in drones compared to queens and workers^17^. Furthermore, drones’ mandibular glands begin to degenerate at an age of 9 days, i.e. when they start leaving the hive, first for orientation flights then for nuptial flights, and before they are fully sexually mature^44, 49, 69^. Other possibilities include antennal glands which are functional in drones^70^ or labial glands which were identified as the source of male-produced attractive components in several bumble bee species^2^. A possible attraction of drones and virgin queens to these glandular sources needs to be tested behaviourally. Then, the identification of active components will require thorough chemical analyses followed by attraction bioassays. Our walking simulator may constitute an ideal tool for testing candidate attractive molecules. The drone-attractive odour is probably a mixture of volatiles. Within this mixture, particular volatiles may be the real attractants, and while our working hypothesis is that the same compounds probably act as both drone and queen attractant, it is possible at this stage that drone and queen attraction are mediated by different molecules. There is also the possibility that the complex blend of drone-produced odours is most effective when all components are present in appropriate ratios.

Although it is important at this stage to remain cautious about whether the drone-produced queen attractant may correspond to a pheromone, one can already wonder how virgin queens’ olfactory system detects and processes this cue. In insects, odorants are detected by olfactory receptors neurons (ORNs) on each antenna which project to a primary olfactory center, the antennal lobe (AL). After local processing in the AL, olfactory information is conveyed to higher-order brain centers, the mushroom bodies (MB) and the lateral horn (LH). Whereas the drone olfactory system is specially adapted for the detection and processing of mating-relevant olfactory cues, with more numerous ORNs and the existence of 4 hypertrophied glomeruli (termed macroglomeruli) in the AL, such adaptations are less obvious in the female olfactory system^71, 72^. AL organization in honeybee queens is quite similar to that in workers, with a slightly lower number of glomeruli^73, 74^. However, one particularly conspicuous glomerulus (corresponding to T1-44 in workers) is the largest in both workers and queens, but its volume relative to the rest of the AL is higher in queens than in workers^72, 75^. For these reasons, it has been hypothesised that this glomerulus could represent a queen macroglomerulus^73^. An interesting hypothesis would be that this putative queen macroglomerulus is dedicated to the processing of a drone-produced pheromone and plays a role in the olfactory orientation toward drone congregations. If behaviourally-active candidate molecules are identified, it should be relatively straightforward to test the activation of the queen MG using *in vivo* optical imaging of the queen brain (as done in workers^76–78^, or drones^79^). One should however keep in mind, that many honey bee pheromones do not seem to be processed through dedicated circuits (labelled lines) but are represented as combinatorial activity from several glomeruli^78^. Accordingly, the queen olfactory system may thus have the ability to process more pheromone stimuli than can be inferred by the observation of a single macroglomerulus. The finding that virgin queens are attracted to odour stimuli from drones thus opens new and fascinating research avenues for understanding the neural basis of sexual communication in insects.

## Methods

### Insects

Walking simulator experiments were performed on queen and worker honeybees (*Apis mellifera*), using groups of drones or of workers as stimuli. All individuals were collected and used during the reproductive season, between June and September. Drones were obtained either from the CNRS campus in Gif-sur-Yvette or from a nearby apiary in Bullion (France). Virgin queens were produced at the CNRS campus (Gif-sur-Yvette) or by a local beekeeping company (Beeopic, Buc, France). Workers were obtained from hives at the CNRS campus.

#### *Focal animals*

Queens and workers which were tested in the walking simulator were age-controlled. They were placed just after emergence in a plexiglas cage^80^ containing a piece of wax comb and providing honey and water *ad libitum*. They were kept in a warm (34 °C) and humid incubator for 7–15 days before the experiments started. A minimum age of 7 days was especially important to obtain sexually mature virgin queens, as honey bee queens usually start leaving the hive for nuptial flights at the age of 7 days^18, 81^.

#### *Stimulus animals*

Stimulus drones were caught at the hive entrance in the afternoon, when they departed for or returned from nuptial flights. Stimulus workers were captured likewise at the hive entrance. Drones and workers were caught on the day of the experiment or on the day before and were placed in a plexiglas cage with honey and water *ad libitum* until used in the experiment.

To avoid a possible impact of genetic relatedness, focal and stimulus subjects always came from different colonies. Queens and drones kept in cages were always accompanied by 10–15 workers to ensure that they remained in good physiological conditions and were fed regularly. All experiments were performed in the afternoon, from 14:00 to 19:30, a time period that is consistent with queens’ and drones’ nuptial behaviour.

### Experimental setup

#### Walking simulator

To test bee’s odour preferences, we used the walking simulator developed by Brandstaetter *et al*.^50^, based on previous insect locomotion compensator systems^82–84^ (Fig. 1). The setup consists of an air-supported ping-pong ball (Cornilleau Competition, Breteuil, France; diameter: 40 mm; weight: 2, 7 g), on which a tethered honey bee was allowed to freely walk in any virtual direction by turning the ball below it. As a ball holder, we used a custom-made Plexiglas block with a hemispherical cavity slightly larger than the ping-pong ball. An air inlet at the bottom of the cavity allowed the ball to float on an air cushion. Air flow was precisely controlled using a pressure regulator (Air Liquide REC BS 50-1-2, Paris, France) and was filtered using activated charcoal (Sigma-Aldrich Norit RB1, Steinheim, Germany). An air extractor was placed behind the bee to avoid any odour contaminations of the setup.

#### Recording

To record ball movement, two highly-sensitive optical sensors from laser mice were used (Logitech G500, Morges, Switzerland: resolution: 5700 dpi, signal rate: 1000 Hz). They were attached to the Plexiglas block at the horizontal equator of the ball and at a relative angle of 90° to each other. The body axis of the insect was always precisely aligned at an angle of 45° with respect to both mouse sensors. Mouse signals were integrated and recorded via custom-written software written in LabView2011 (National Instruments, Nanterre, France) using ManyMouse to separately handle the signals of both mouse sensors (source code by Ryan C. Gordon; http://icculus.org/manymouse). From the recorded ball movements, custom-written software directly calculated the bee’s walking path, and produced throughout the experiment several parameters such as its walking speed, turning direction and virtual heading.

#### Bee fixing

Each bee was shortly anesthetized on crushed ice and a very small insect needle (minutens 3.20, Ento Sphinx, Pardubice, Czeck Republic) was glued to its thorax using low-temperature melting wax (Deiberit 502; Schöps and Dr. Böhme, Goslar, Germany), or using UV-reactive glue (3 M ESPE Sinfony dentique opaque 3, Cergy-Pontoise, France) and a curing light (Woodpecker LED. B, Guilin, Guangxi, PR China). After at least 5 min of recovery time, the tethered bee was placed on the walking simulator. In this situation, the bee could only walk by turning the ball but could not fly.

#### Odour stimulation

We applied the protocol from the second experiment in Brandstaetter *et al*.^50^. Walking bees were subjected to a continuous airflow in which odour stimulations could be applied. It was delivered via a glass tube (inner diameter: 7 mm) directly in front of the bee at a distance of 20 mm, directed to its antennae (Fig. 1). The air flow consisted of a main continuous air flow (1 L/h) and a secondary air flow (0.2 L/h), which were filtered by activated charcoal (Sigma-Aldrich Norit RB1) and regulated by flow-meters (Brooks Instrument Model 1355E Sho-rate, R-2-15-D and R-2-15-AAA respectively, Hatfield, PA, USA). An odour stimulation could be applied using computer controlled magnetic valves (Lee LFAA1200118H, Voisins Le Bretonneux, France; controlled via a BMCM R8 relay and USB-PIO, Maisach, Germany), switching the secondary continuous airflow from an empty Pasteur pipette to an odour pipette attached to a vial containing stimulation animals. Due to the fast switching magnetic valves between control pipette and odour pipette, total air flow in front of the bee was held at a constant rate of 1.2 L/h. As olfactory stimulation, we used the odour bouquet from groups of 10 living drones or 10 living workers. Each group was placed in a 100 mL vial which was connected to the odour Pasteur pipette of the secondary air flow (see Fig. 1).

#### Experimental Protocol

Each experiment consisted of two periods of 5 minutes each. First, bees received a 5 min accommodation to the experimental setup, during which it could freely walk on the ball (‘before’ phase). After that time, full control over odour stimulation was given to the bee during 5 minutes (‘during’ phase). To this end, the ball was virtually divided into 4 quadrants, and one quadrant was pseudo randomly designated as the odour quadrant. Odour stimulation was activated whenever the bee was heading toward the virtual odour quadrant (Fig. 1). Thus, the tested bee received a clear feedback from its own behaviour (closed loop). Because turning the ball is an easy task for the bee, we can evaluate whether it preferred to receive odour stimulation or not. Following our preliminary experiment testing workers with an appetitively learned odorant (Supplementary Experiment 1), attraction can be measured by the time spent and the distance walked by the insect in the odour quadrant relative to the other quadrants. To signal to the bee the presence of an odour cue in the setup, a 1 sec odour pulse was given at the beginning of the stimulus control phase. All experiments were performed in complete darkness under an opaque cage protecting the setup from any stray light and undesired air currents.

### Data analysis and statistics

To ensure that the results reflected the behaviour of fit, well-positioned and closed-loop aware bees, three selection criteria were used: 1) Mobility: unfit bees, i.e. bees that walked less than 200 mm during the first 5 min, were excluded; 2) Lateral bias: bees that turned more than 7200° (i.e. 20 full turns) in any one direction during the first 5 min were also excluded, as they were either fixed in an inadequate position on the ball or were too strongly lateralized. 3) Closed loop: because the experiment aims to measure bee’s behavioural choice to receive or not the odorant stimulation, bees that never experienced their own control over odour delivery cannot be kept^50^. Thus, individuals that never toggled the odour ON or OFF through their own behaviour during the stimulus control (i.e. ‘during’) phase, i.e. bees that never switched from an odour quadrant to a non-odour quadrant (and vice versa), were excluded. Overall, 5 out of 24 workers (21%) and 7 out of 45 queens (16%) were discarded.

Ball movement data were acquired at 5 Hz frequency, so that bees’ virtual position could be calculated every 200 ms, giving access to its virtual heading and the distance covered. In the figures, we represented the percentage of the time spent and of the distance travelled during each phase, either in a circular graph by 15° sectors (Figs 2A,C–4A,C), or in a boxplot for the odour quadrant and the average of the 3 other quadrants (Figs 2B,D–4B,D). To detect a significant orientation of bees toward the odour stimulus, time spent and distance travelled in the odour was compared to the average of the 3 other quadrants using Wilcoxon matched pairs tests. We compared the distance travelled by insects in the different experiments using a Kruskal-Wallis test. Pairwise comparisons were performed using Dunn’s test, which includes a correction for multiple comparisons. The Wilcoxon test was used to compare travelled distance between phases within each experiment. Graphs were plotted using OriginPro 8.5 (OriginLab, Northampton, MA, USA) and statistical analyses were performed using Statistica 8.0 (StatSoft, Tulsa, OK, USA).

## Electronic supplementary material

Supplementary Experiment 1
