# Supplementary material for: Virgin queen attraction toward males in honey bees
Source: Sci Rep. 2017 Jul 24;7:6293. doi: 10.1038/s41598-017-06241-9 (PMC5524964; doi:10.1038/s41598-017-06241-9)
Supplement: Supplementary file 1 — Supplementary Experiment 1 [file 41598_2017_6241_MOESM1_ESM.pdf]

Supplementary material

Title: Virgin queen attraction toward males in honey bees

Authors: Florian Bastin, Hanna Cholé, Grégory Lafon and Jean-Christophe Sandoz

Supplementary Experiment 1

In this study, we used a recently developed protocol to measure odour preferences in a walking simulator setup in honey bees[1]. In this supplementary experiment, we aimed to further validate its ability to measure a significant attraction using a situation that is known to induce clear attraction from honey bees. For this purpose, we performed an olfactory information transfer experiment in which bees were first conditioned appetitively to associate an initially neutral odorant with a sucrose reward (PER conditioning) and then tested on the walking simulator to assess the attractiveness of the learned odorant. In previous studies a clear and significant odour attraction was observed after PER conditioning in a variety of situations: walking in a four-armed olfactometer[2], walking in a Y-maze[3] or flying in a wind tunnel[4].

The experimental procedure applied for PER conditioning was the standard one used in previous studies on olfactory learning in honeybees[5-7]. Bees were subjected to 5 conditioning trials with 10 min inter-trial intervals, during which an odour CS (1-nonanol) was associated with a sucrose US (see detailed methods below). In parallel to the conditioned bees, a control group underwent the same procedure but without odour delivery. Both groups thus received the same amount of sucrose solution, so that no energy difference may explain possible behavioural differences on the ball. Conditioned bees efficiently learned the odorant that preceded sucrose solution in the course of training (Cochran Q Test: $Q = 153.386$, $p < 0.001$, $N = 46$, Suppl. Fig.1). At the last conditioning trial, 100% of conditioned workers responded to the CS. Bees that showed robust learning, i.e. that responded with a PER to the three last conditioning trials were kept for the walking simulator tests.

Two hours after the end of conditioning, the olfactory preferences of bees were tested in our walking simulator. After a stimulation-free accommodation phase of 5 min (henceforth termed 'before' phase), stimulus control was granted to the workers for 5 min (henceforth termed 'during' phase). The ball was divided in four virtual quadrants, one of which was pseudo-randomly designated as the odour quadrant. In the 'during' phase, whenever the worker was heading toward the odour quadrant, odour stimulation with the CS was activated and delivered directly in front of the bee to its antennae. During the accommodation phase (‘before’), the time spent and the distance travelled by conditioned workers (Suppl. Fig.2) were similar between the odour quadrant and the non-odour quadrants (Wilcoxon test:

Z_{time}=1.09, p_{time}=0.29; $Z_{distance}$=1.61, $p_{distance}$=0.11, N=19). However, in the odour stimulation phase ('during'), conditioned workers spent significantly more time (Suppl. Fig 2A,B) and travelled a longer distance (Suppl. Fig 2C,D) in the odour quadrant compared to the non-odour quadrants (Z_{time}=3.10, p_{time}=0.002; $Z_{distance}$=2.58, $p_{distance}$=0.010). In control bees, the situation was different. These unconditioned bees spent the same amount of time and travelled the same distance in the odour and in the non-odour quadrants, both before (Suppl. Fig 3, Z_{time}=1.09, p_{time}=0.28; $Z_{distance}$=1.73, $p_{distance}$=0.08, N=19) and during the odour stimulation phase (Suppl. Fig 3, Z_{time}=0.24, p_{time}=0.81; $Z_{distance}$=0.00, $p_{distance}$=1.00). These experiments clearly show that in the walking simulator, bees were attracted to an odour which had acquired a positive (attractive) value through learning. They spent more time and travelled a longer distance in the odour quadrant. This experiment thus validates the functionality of our experimental setup for studying odour preferences of honeybees under controlled experimental conditions.

Methods

Honeybee workers (*A. mellifera*) were caught from hives on the CNRS campus in Gif-sur-Yvette (France). The bees were caught in the morning, chilled on ice and were then harnessed individually in metal holders, leaving their antennae and mouthparts free.

PER conditioning

Bees were subjected to absolute conditioning with 5 PER conditioning trials and an inter-trial interval of 10 min. For the odour stimulation, 5 microliters of pure 1-nonanol (Sigma Aldrich) were applied to a 1 cm^2 piece of filter paper, which was placed into a Pasteur pipette. The odour source was connected to a pump, enabling the constant circulation of an air flow of 52.5 mL/s. This flow, composed of a principal air flow of 50 mL/s and a secondary flow of 2.5 mL/s, was directed to the bee through a glass tube (0.5 cm diameter), at a distance of 2 cm. The secondary airflow could be directed to one of two subcircuits (one containing the 1-nonanol, and another without any odorant) before being reinjected into the main airflow. Most of the time, air flowed through the odourless subcircuit.

Before each trial, bees were positioned for 20 s in the airflow to familiarize them with the mechanical stimulation. Then, the olfactory CS was applied, inducing a switch of the secondary flow to the 1-nonanol subcircuit for 6 s. Three seconds after CS onset, the US was applied for three seconds, first to the antennae and then to the proboscis, at which point the bee could lick the sucrose solution. An air extractor, placed behind the bee prevented odorant

accumulation. Since this experiment aimed at recording bees' behaviour on the walking simulator when presenting an odorant that had acquired a positive value for them, only bees that showed a robust learning performance during PER conditioning were conserved. All bees kept in the conditioned group thus responded with a proboscis extension to the CS at least in the three last trials. In parallel to the conditioned bees, a control group received the same procedure expect that no odour CS was delivered (only sucrose reward).

Walking simulator-attraction test

Between 2 and 5 hours after PER conditioning, workers were tested individually in the walking simulator. The same setup and protocol as the one described in the main text were used, except that here a synthetic odorant (1-nonanol) was used as stimulus. The odour source consisted of 10 µl of 1-nonanol onto a filter paper strip (1 cm^2) placed in a Pasteur pipette. For odour stimulation in the 'during' phase, in order to avoid possible strong olfactory adaptation that may be caused by potentially prolonged periods of stimulation (if the animal remains in the odour quadrant), odour presentation was pulsed with an on/off phase of 100 ms each (see synthetic odour experiments in ref. 1).

References:

1. Brandstaetter, A. S., Bastin, F. & Sandoz, J. C. Honeybee drones are attracted by groups of consexuals in a walking simulator. *J. Exp. Biol*. **217**, 1278-1285 (2014).

2. Sandoz, J. C., Laloi, D., Odoux, J. F. & Pham-Delegue, M. H. Olfactory information transfer in the honeybee: compared efficiency of classical conditioning and early exposure. *Anim. Behav.* **59**(5), 1025-1034 (2000).

3. Carcaud, J., Roussel, E., Giurfa, M. & Sandoz, J. C. Odour aversion after olfactory conditioning of the sting extension reflex in honeybees. *J. Exp. Biol.* **212**, 620-626 (2009).

4. Chaffiol, A., Laloi, D. & Pham-Delegue, M. H. Prior classical olfactory conditioning improves odour-cued flight orientation of honey bees in a wind tunnel. *J. Exp. Biol.* **208**, 3731-3737 (2005).

5. Bitterman, M. E., Menzel, R., Fietz, A. & Schäfer, S. Classical conditioning of proboscis extension in honeybees. *J. Comp. Psychol.* **97**(2), 107-119 (1983).

6. Giurfa, M. & Sandoz, J. C. Invertebrate learning and memory: Fifty years of olfactory conditioning of the proboscis extension response in honeybees. *Learn. Mem.* **19**(2), 54-66 (2012).

7. Matsumoto, Y., Menzel, R., Sandoz, J. C. & Giurfa, M. Revisiting olfactory classical conditioning of the proboscis extension response in honey bees: a step toward standardized procedures. *J. Neurosci. Methods* **211**(1), 159-167 (2012).

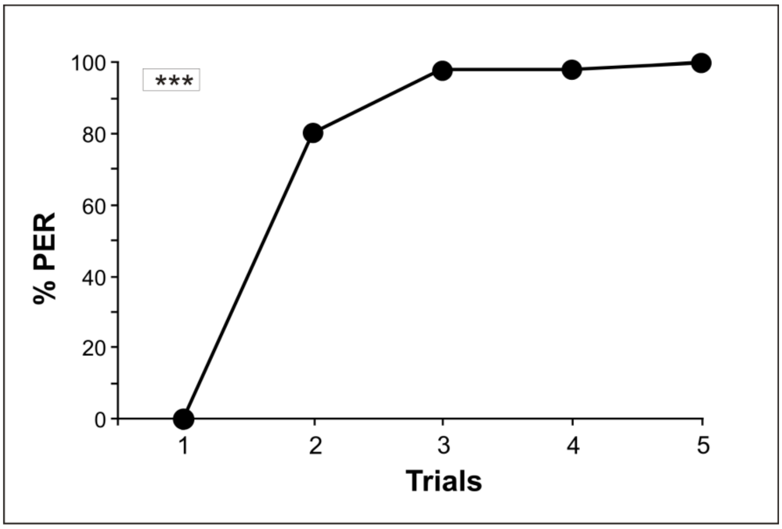

Supplementary Figure 1: **Learning performances of workers in the appetitive conditioning procedure**. Percentage of proboscis extension responses (PER) in bees trained with 1-nonanol reinforced with sucrose solution (N=46) along 5 trials. Workers efficiently learned the odour-reward association, as all workers responded to 1-nonanol at the fifth trials. ***: $p<0.001$, Cochran Q Test.

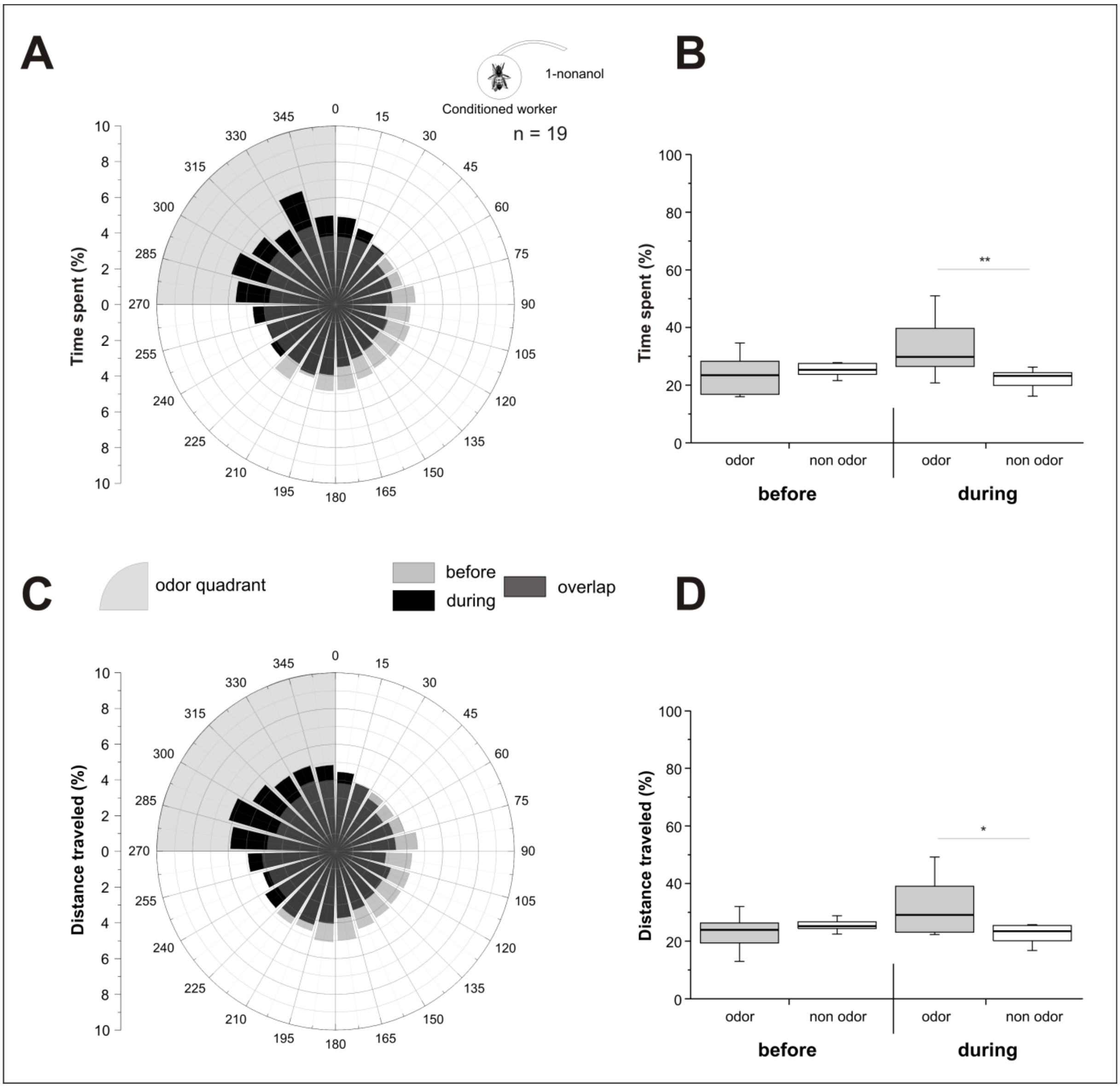

Supplementary Figure 2: **Conditioned workers' response to an appetitively learned odour**. Conditioned workers' behaviour on the walking simulator, when stimulated with 1-nonanol (conditioned stimulus), which acquired a positive hedonic value through learning. **A,C)** Circular histograms showing the percentage of time spent (A) or of distance travelled (C) by conditioned workers according to 15° sectors, with the odour quadrant being represented on the upper left (grey area). Light grey bars represent the 5 min before odour stimulation ('before'), black bars represent the 5 min during stimulation ('during'), and hence, dark grey bars show the overlap of the two phases. **B,D)** Histograms of the percentage of time spent (B), or of distance travelled (D) by conditioned workers in the odour quadrant (gray box) and on average in the three odourless quadrants (white box) before and during odour stimulation. *: $p<0.05$, **: $p<0.01$, Wilcoxon matched pairs tests.

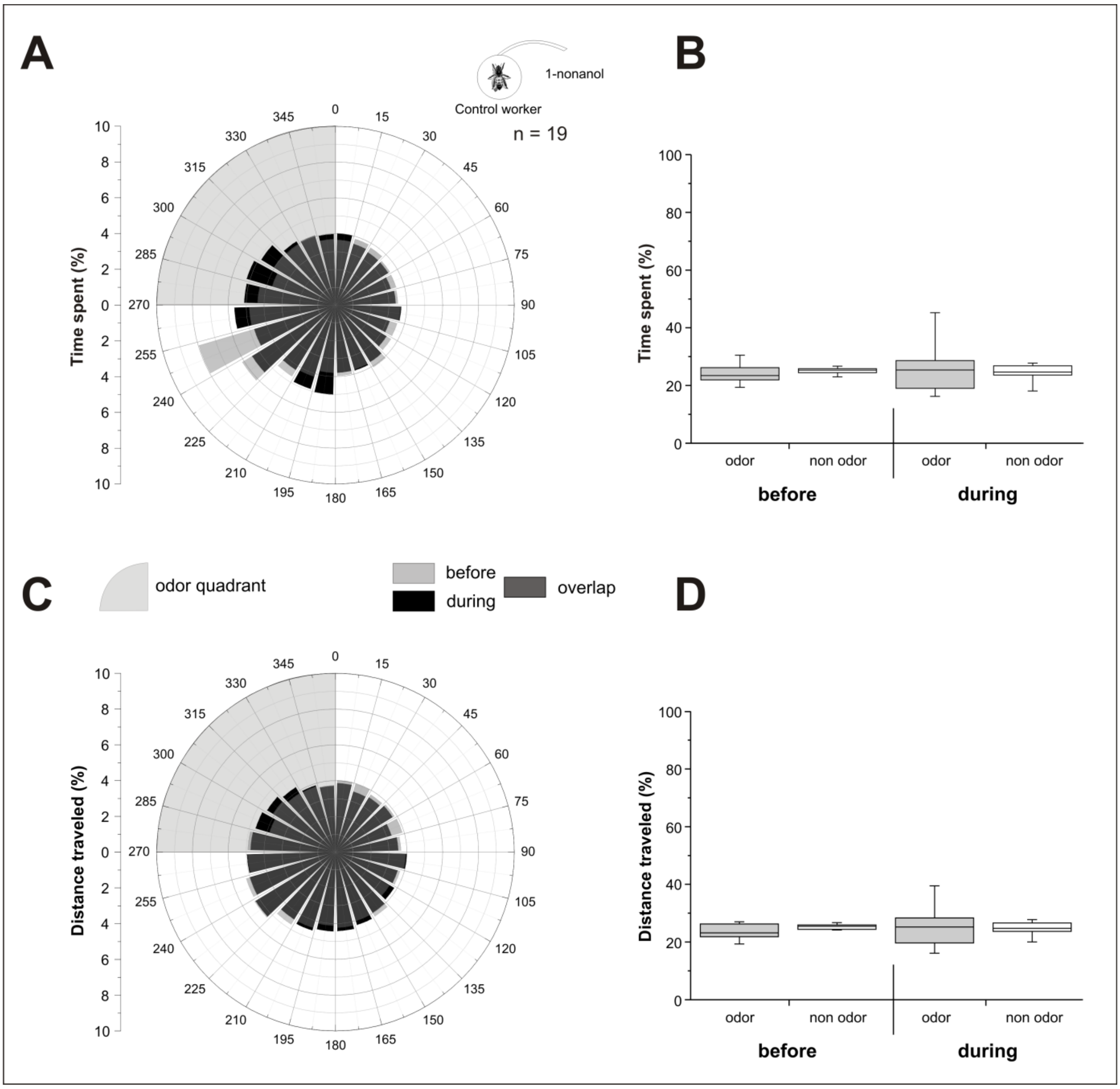

Supplementary Figure 3: **Control workers' response to a novel odour**. Unconditioned workers' behaviour on the walking simulator, when stimulated with 1-nonanol, which is a novel odour for these bees. **A,C)** Circular histograms showing the percentage of time spent (A) or of distance travelled (C) by unconditioned workers according to 15° sectors, with the odour quadrant being represented on the upper left (grey area). Light grey bars represent the 5 min before odour stimulation ('before'), black bars represent the 5 min during stimulation ('during'), and hence, dark grey bars show the overlap of the two phases. **B,D)** Histograms of the percentage of time spent (B), or of distance travelled (D) by control workers in the odour quadrant (gray box) and on average in the three odourless quadrants (white box) before and during odour stimulation. NS, Wilcoxon matched pairs tests.

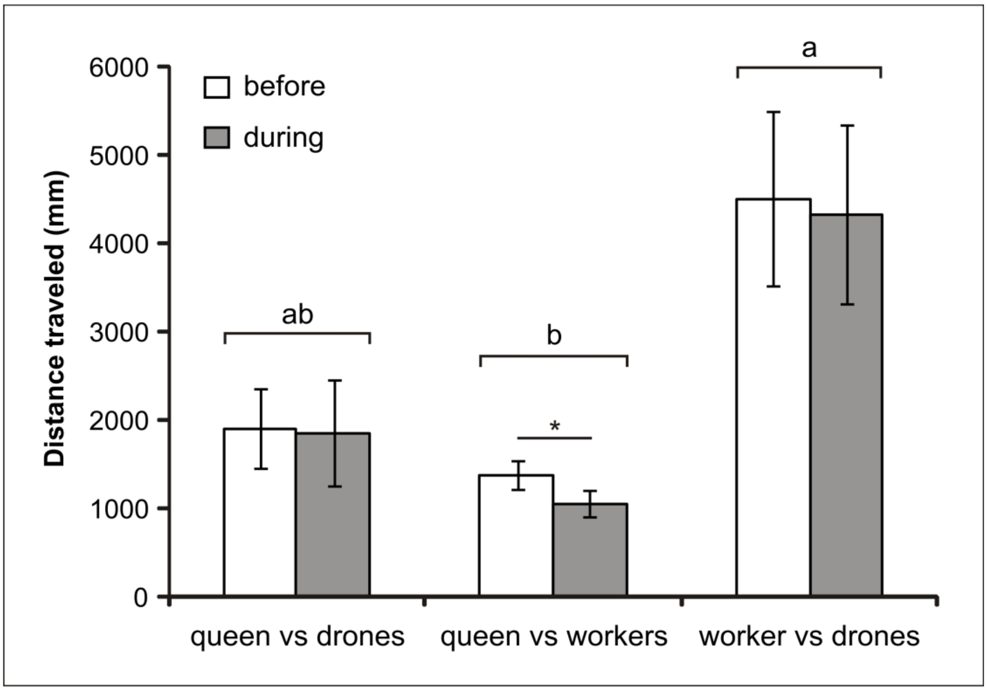

Supplementary Figure 4: Total distance travelled in Experiments 1-3. Distance (in mm) travelled on the walking simulator, before (5 min) and during odor stimulation (5 min), in each experiment: 1 (queen walking with drone odour stimulation), 2 (queen walking with worker odour stimulation), and 3 (worker walking with drone odour stimulation). White bars represent the phase before' odour stimulation, whereas grey bars represent the phase 'during' odour stimulation. Letters indicate significant differences between the experiments (Kruskal-Wallis test, followed by a Dunn's multiple comparisons test as post-hoc). *: $p<0.05$, comparison between phases, Wilcoxon matched pairs tests.
